# Molecular Expression Differences in Specific Blood Mononuclear Cell‐Types Identify Patients With AL Amyloidosis

**DOI:** 10.1111/jcmm.70850

**Published:** 2025-09-22

**Authors:** David Kaplan, Eric Christian, Jason Valent, Faiz Anwer, Sandra Mazzoni, Christy Samaras, Louis Williams, Megan Nakashima, Shahzad Raza, Mazen Hanna, Hillard M. Lazarus, Jack Khouri

**Affiliations:** ^1^ CellPrint Biotechnology Cleveland Ohio USA; ^2^ Department of Pathology Case Western Reserve University Cleveland Ohio USA; ^3^ Amyloidosis Center, Taussig Cancer Center, Cleveland Clinic Foundation Cleveland Ohio USA; ^4^ Department of Pathology Cleveland Clinic Foundation Cleveland Ohio USA; ^5^ Department of Medicine Case Western Reserve University Cleveland Ohio USA

**Keywords:** AL amyloidosis, blood‐based biomarker, cell‐specific molecular expression, diagnostic assay, myeloma

## Abstract

The diagnosis of AL amyloidosis is often challenging due to its systemic nature and heterogeneous clinical presentation. Current serological biomarkers for diagnosis and monitoring are not optimal. We have considered the possibility that mononuclear cell‐type specific molecular expression can be used to develop blood‐based biomarkers to diagnose and monitor patients with AL amyloidosis. Peripheral blood monocytes and CD4^+^ T cells from patients with documented AL amyloidosis or myeloma without amyloidosis were assessed by enhanced flow cytometric analysis for expression levels of 20 analytes chosen for the possibility that their expression levels may lead to diagnostic assays and biomarkers. We found definitive expression level differences for brain‐derived neurotrophin factor (BDNF), calmodulin, and phospho‐TBK1 in CD4^+^ T cells and for phospho‐GSK3β in monocytes. Logistic regression and ROC analysis showed that BDNF in CD4^+^ T cells and heme oxygenase 1 in monocytes significantly distinguished between patients with myeloma versus patients with AL amyloidosis (AUC = 0.75). Additionally, we discovered remarkable differences in intermolecular associations between the samples from the two patient groups, suggesting the involvement of specific pathogenetic pathways. Our results demonstrate that mononuclear cell‐type specific molecular expression may be useful for developing a diagnostic assay and biomarkers for patients with AL amyloidosis.

**Abbreviations**
BDNF: brain‐derived neurotrophic factor
HO1: heme oxygenase
rdC: restricted dimensional cytometry
SQST: squestasome

## 1 Introduction

The diagnosis of AL amyloidosis is challenging due to its rarity and highly variable clinical presentation, often leading to delayed diagnosis and worse outcomes [1, 2, 3]. The pathogenesis is driven by aberrant free immunoglobulin light chain deposits in different organs, leading to organ dysfunction, morbidity, and mortality [4].

Diagnosis of AL amyloidosis relies on the demonstration of amyloid fibril deposits on tissue biopsy, which could be obtained from target organs or surrogate tissues such as fat pad and bone marrow, in addition to the presence of a plasma cell clone. Securing tissue biopsy can be challenging due to accessibility issues, biopsy‐related complications, and suboptimal sampling technique, especially for fat pad analysis. The backbone of diagnosis and response evaluation in AL relies heavily on serum free light chains, which have been instrumental in disease detection and monitoring. However, they have several limitations, including the inability to isolate toxic light chains or detect low levels of toxic light chains, as well as the lack of reliability in the setting of kidney dysfunction.

Therefore, there is a need for more sensitive and reliable diagnostic and monitoring assays. Recently, mass spectrometry has emerged as a more sensitive and specific assay with significant implications for diagnosis and response assessment in AL amyloidosis [5]. We considered the possibility that the presence of amyloid deposits in tissues may be reflected by distinct changes in the molecular expression of specific types of mononuclear cells circulating in the peripheral circulation. Mononuclear cells possess receptors for components in the tissue depositions of AL amyloidosis such as serum amyloid P and complement [6, 7, 8, 9, 10]. Consequently, receptor‐triggered signalling initiated by amyloid deposits may result in characteristic changes in molecular expression levels and patterns and may be detected with a sensitive single‐cell technology. In this way, blood mononuclear cells may be used as accessible and sensitive monitors of the presence of the pathological AL amyloidosis deposits in tissues.

We have previously developed a powerful flow cytometric platform with 10–100 fold enhanced sensitivity of detection over standard practices and restricted dimensionality (rdC) that we have applied to analyse cell‐type specific molecular expression with extraordinary precision [11]. Our platform has been used previously to identify blood‐based mononuclear cell‐type specific biomarkers for patients with chronic lymphocytic leukaemia, bipolar disorder, and autologous haematopoietic cell transplantation [11, 12, 13, 14, 15, 16, 17, 18, 19, 20, 21, 22, 23, 24, 25, 26, 27]. Consequently, we tested the possibility that specific patterns of molecular expression in defined mononuclear cell‐types could be identified that are characteristic of AL amyloidosis and that could distinguish patients with AL amyloidosis from patients with myeloma and no amyloidosis.

## 2 Materials and Methods

### 2.1 Cell Donors

We collected peripheral blood samples from patients with AL amyloidosis (*n* = 27) and patients with myeloma (*n* = 40). All samples originated at the Amyloidosis Center at the Cleveland Clinic Foundation Taussig Cancer Center and were obtained after Institutional Review Board approval. Informed consent was obtained prior to participation. The samples were obtained between 9:00 AM and 1:00 PM and were processed within four hours of venipuncture.

### 2.2 Cells

The mononuclear cell fraction was isolated via discontinuous gradient separation over ficoll/hypaque. The mononuclear cells were suspended in medium containing dimethyl sulfoxide, viably frozen, and stored in liquid nitrogen. The laboratory personnel were blinded to the clinical data associated with the samples. The code was not broken until the expression level dataset was finalised.

### 2.3 Reagents

The primary antibodies used in the study were rabbit monoclonals obtained from commercial sources. Primary antibodies were obtained from Cell Signalling Technology (Danvers, MA) with specificities for phospho‐Atg14 ser29 (cat#92340), phospho‐Bcl2 ser70 (#2827), phospho‐cJun ser73 (#3270), phospho‐RelA ser536 (#3033), phospho‐p38 MAPK Thr180/Tyr182 (#9215), phospho‐Erk1/2 thr202/tyr204 (#4370), phospho‐GSK3β ser9 (cat#5558), phospho‐RIP ser166 (cat#44590), phospho‐sequestasome (phospho‐SQST) ser379 (#16177), phospho‐Shp2 tyr580 (#5431), phospho‐STING ser366 (cat#40818), phospho‐TBK1 ser172 (cat#5483), phospho‐ULK1 ser757 (#14202), phospho‐ZAP70 tyr319 (#2717), and Syk (#13198). Primary antibodies were obtained from Abcam (Boston, MA) with specificities for brain‐derived neurotrophic factor (BDNF; #ab108319), calmodulin (#ab45689), HMGB1 (#ab79823), and Vav (#ab40875). Primary antibody was obtained from BosterBio (Pleasanton, CA) with specificity for heme oxygenase 1 (HO1; #M00253).

### 2.4 Enhanced Flow Cytometric Analysis

Cells were thawed, stained with antibodies to the CD4 lineage marker, fixed, permeabilised, and stained for the expression of selected analytes with signal amplification by *CellPrint*. The anti‐CD4 co‐staining antibody labelled with DyLight650 was obtained from BioLegend (San Diego, CA). Signal amplification was accomplished as previously described [11, 12, 13, 14, 15, 16, 17, 18, 19, 20, 21, 22, 23, 24, 25, 26, 27]. Briefly, after labelling with the primary rabbit monoclonal antibodies, the cells were stained with anti‐rabbit immunoglobulin labelled with horseradish peroxidase and then exposed to fluorescein tyramide in the presence of hydrogen peroxide. Each tube included a single lineage marker and a single pathway analyte rabbit monoclonal which was amplified. The cells were assessed for fluorescent intensity on a BD Accuri flow cytometer, and the results were analysed with FloJo software.

### 2.5 Statistical Analysis

Bivariate correlations, *t* tests, logistic regression, and ROC analysis were performed with SPSS and Excel software. The comparison of correlation coefficients was accomplished after *r*‐to‐*z* transformation online at vassarstats.net/rdiff.html.

## 3 Results

### 3.1 Patient Characteristics

Myeloma and AL amyloidosis patients donated peripheral blood for our study. The patients are described in Table S1. Both the proportions of gender and race differed between the two groups of patients. Also, the specific therapeutic agents used differed for the treated patients with myeloma and AL amyloidosis.

### 3.2 Data Quality

Mononuclear cells were analysed for the expression of 20 distinct analytes. The data obtained gave sharp peaks of expression. Figure S1 shows representative results for CD4^+^ T cells. The average number of CD4^+^ T cells analysed per peak was 6222. The sharp peaks indicate that the median value is a reasonably precise indicator for the population and that cellular subsets did not represent significant variance in expression levels. The histogram for HMGB1 expression shows some variance. In our analysis, only the sharp, high‐expression peak was included in the analysis. The large number of events included in the analysis ensures high precision in the values obtained.

Results for monocytes showed similar patterns with sharp peaks of expression. For monocytes, the average number of cells analysed per peak was 7245.

### 3.3 Molecular Expression Level Differences

**FIGURE 1:**
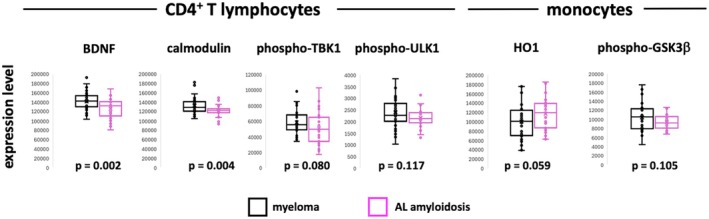
Expression level differences in CD4^+^ T cells and monocytes from patients with myeloma and patients with AL amyloidosis. The expression levels of BDNF, calmodulin, and phospho‐TBK1 in CD4^+^ T cells and HO1 and phospho‐GSK3β in monocytes are shown for 40 myeloma patients without amyloid deposits and for 27 AL amyloidosis patients. The *p* values for the *t* tests are provided at the bottom of each panel.

Figure 1 shows the analytes that demonstrated differential expression in either CD4^+^ T cells or monocytes between patients with AL amyloidosis and CD4^+^ T cells from patients with myeloma without amyloid deposits. The mononuclear cells from AL amyloidosis patients had lower expression levels than the cells from myeloma patients for BDNF, calmodulin, phospho‐TBK1, and phospho‐ULK1 in CD4^+^ T cells and phospho‐GSK3β in monocytes, but higher levels of HO1 in monocytes. None of the other seventeen molecules showed differences in expression between cells from patients with AL amyloidosis compared to cells from myeloma patients, with *p* values for *t* tests greater than 0.2 (Figures S2 and S3).

**FIGURE 2:**
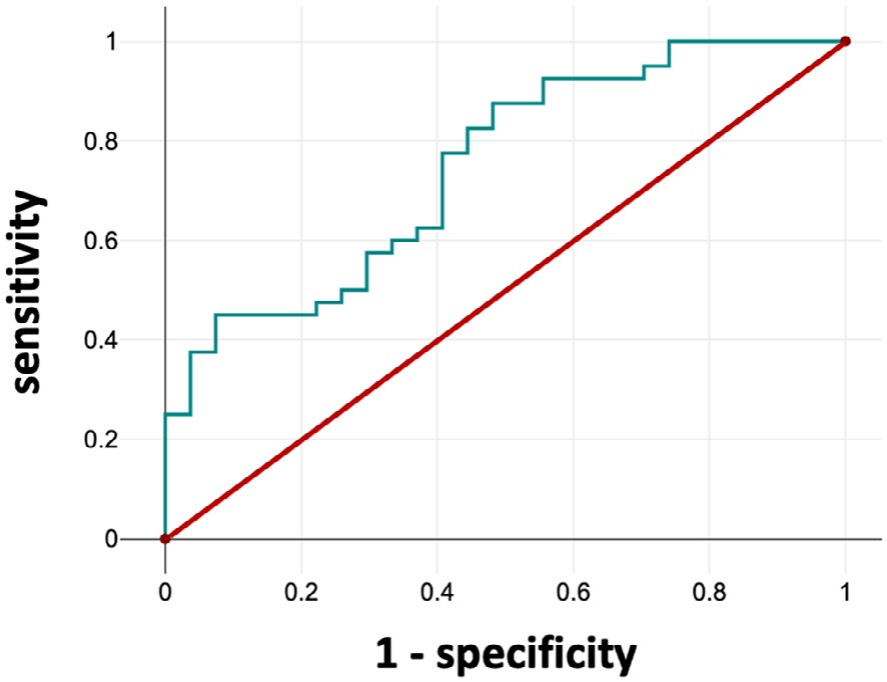
ROC analysis predicting amyloidosis. BDNF in CD4^+^ T cells and HO1 in monocytes were included in a ROC analysis to distinguish patients with myeloma and no amyloidosis from patients with AL amyloidosis. AUC = 0.75 (95% confidence interval = 0.66–0.86). *p* < 0.001.

Logistic regression was used to assess the capacity of the expression levels of these analytes to distinguish between patients with myeloma and no amyloid deposits and patients with biopsy‐demonstrated amyloid deposits. BDNF in CD4^+^ T cells and HO1 in monocytes were included in the model individually. The results were similar, with excellent negative predictive value for determining patients without amyloid deposits, but poor positive predictive value for identifying patients with AL amyloidosis (Table 1). By including both of these parameters in the model, we found a marked improvement in the positive predictive value compared to either of the models with single parameters included (Table 1). For this model, the sensitivity for detecting AL amyloidosis is 60%, and the specificity is 76%. The ROC analysis with both parameters (AUC = 0.75) is shown in Figure 2.

**TABLE 1:** Logistic regression models classifying patients with AL amyloidosis and myeloma.

		**Model: BNDF in CD4** ^**+**^ **T cells** ^a^		**Predictive value (%)**
		**AL amyloidosis**	**Myeloma**	
**Clinical determination** ^d^	**AL amyloidosis**	11	16	41
	**Myeloma**	7	33	83
		**Model: HO1 in monocytes** ^b^		**Predictive value (%)**
		**AL amyloidosis**	**Myeloma**	
**Clinical determination** ^d^	**AL amyloidosis**	10	17	37
	**Myeloma**	7	33	83
		**Model: BNDF in CD4** ^**+**^ **T cells + HO1 in monocytes** ^c^		**Predictive value (%)**
		**AL amyloidosis**	**Myeloma**	
**Clinical determination** ^d^	**AL amyloidosis**	15	12	56
	**Myeloma**	8	32	80

^a^ The *p* value for CD4^+^ T cell BDNF in the model was 0.006.

^b^ The *p* value for monocytic HO1 in the model was 0.064.

^c^ The *p* values for CD4^+^ T cell BDNF and monocytic HO1 in the combined model were 0.003 and 0.038 respectively.

^d^ Clinical determination was based on physical examination, laboratory results, and tissue biopsy.

### 3.4 Bivariate Correlational Differences

We found significant bivariate relationships because of the high quality of our results and the precision of the expression levels we obtained. For instance, in the CD4^+^ T cells in samples from patients with myeloma or AL amyloidosis, six analytes (phospho‐p38 MAPK, phospho‐RIP, phospho‐Shp2, phospho‐SQST, phospho‐STING, and phospho‐ULK1) demonstrated high levels of bivariate correlations (Figures S4 and S5, Table S2). No differences in correlation coefficients between samples from patients with myeloma versus patients with AL amyloidosis were found after *r*‐to‐*z* transformation.

**FIGURE 3:**
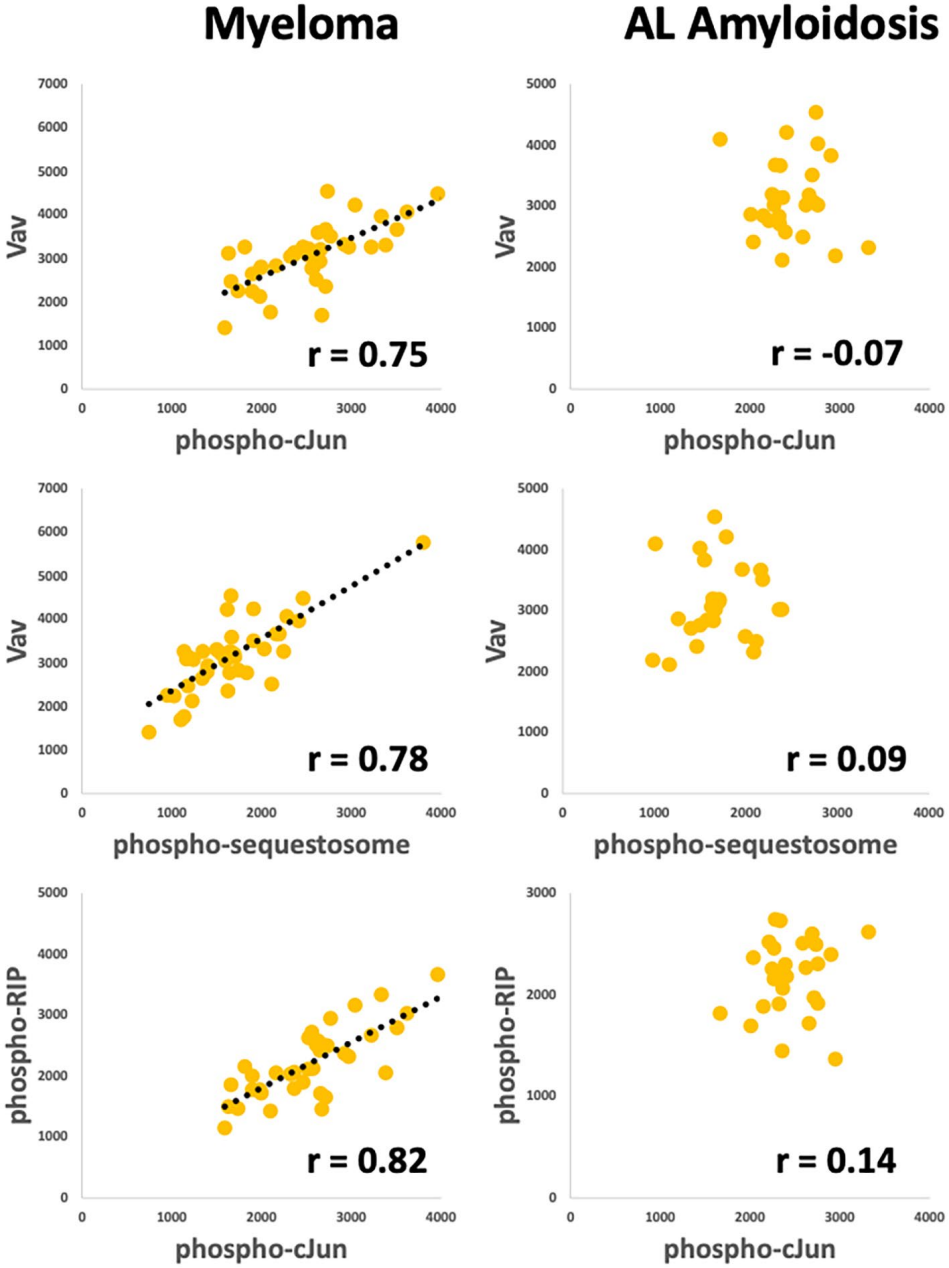
Examples of differences in bivariate correlation in CD4^+^ T cells between samples from myeloma patients and patients with AL amyloidosis. Correlation coefficients are shown in the lower right corner of each panel.

However, not all bivariate correlations are preserved in both sets of patients. Both Vav and phospho‐cJun are strongly correlated with various phosphoantigens as well as with each other in CD4^+^ T cells from myeloma patients, but not in the cells from patients with AL amyloidosis (Tables 2 and 3, Figure 3). Assessing the significance of the different *r* values was accomplished after *r*‐to‐*z* transformation and demonstrated a high likelihood that the *r* values derived from the myeloma patients were distinct from the *r* values derived from the patients with AL amyloidosis.

**TABLE 2:** Bivariate correlations centred on phospho‐cJun expression in CD4^+^ T lymphocytes.

Keystone molecule	Molecular partners	Myeloma		AL amyloidosis		*r* to *z* (*p*)
		*r*	*p*	*r*	*p*	
Phospho‐cJun	Vav	0.75	3 × 10^−8^	−0.07	0.73	0.0001
	Phospho‐RIP	0.82	1 × 10^−10^	0.14	0.49	0.0001
	Phospho‐p38 MAPK	0.77	6 × 10^−9^	0.28	0.16	0.0051
	Phospho‐Shp2	0.77	6 × 10^−9^	0.35	0.07	0.0124
	Phospho‐STING	0.81	2 × 10^−10^	0.14	0.49	0.0002
	Phospho‐SQST	0.79	1 × 10^−9^	0.37	0.06	0.0091
	Phospho‐Atg14	0.73	9 × 10^−8^	0.20	0.32	0.0056

*Note:* Correlation coefficients (*r*) with their associated *p* values are shown. Values of *r* were compared between samples from patients with myeloma and AL amyloidosis after conversion to *z*.

**TABLE 3:** Bivariate correlations centered on Vav expression in CD4^+^ T lymphocytes.

Keystone molecule	Molecular partners	Myeloma		AL amyloidosis		*r* to *z* (*p*)
		*r*	*p*	*r*	*p*	
Vav	Phospho‐cJun	0.75	3 × 10^−8^	−0.07	0.73	0.0001
	Phospho‐RIP	0.86	1 × 10^−12^	0.27	0.17	0.0001
	Phospho‐p38 MAPK	0.68	1 × 10^−6^	−0.07	0.73	0.0006
	Phospho‐Shp2	0.74	5 × 10^−8^	0.06	0.77	0.0007
	Phospho‐STING	0.72	2 × 10^−7^	−0.10	0.62	0.0001
	Phospho‐SQST	0.78	3 × 10^−9^	0.09	0.66	0.0003
	Phospho‐GSK3β	0.71	3 × 10^−7^	0.15	0.46	0.0050
	Phospho‐Erk	0.63	1 × 10^−5^	0.04	0.84	0.0074

*Note:* Correlation coefficients (*r*) with their associated *p* values are shown. Values of *r* were compared between samples from patients with myeloma and AL amyloidosis after conversion to *z*.

Conversely, we found bivariate correlations in the cells from AL amyloidosis patients that were apparent in the CD4^+^ T cells from the myeloma patients (Table S3 and Figure S6). BDNF and phospho‐TBK1 were significantly correlated in CD4^+^ T cells from AL amyloidosis patients but not in myeloma patients. The expression levels of both BDNF and phospho‐TBK1 were elevated in the CD4^+^ T cells from AL amyloidosis patients compared to myeloma patients (Figure 2).

Monocytes demonstrated strikingly similar bivariate correlations between patients with myeloma and AL amyloidosis, with the exception of BDNF as a keystone molecule (Table S4 and Figure S7).

## 4 Discussion

By restricting the dimensionality of cytometric analysis, we have produced highly precise assessments of blood mononuclear cell‐type specific molecular expression levels which can distinguish between patients who are affected by myeloma compared to those with AL amyloidosis. Differences in expression in both CD4^+^ T cells and monocytes were included in a logistic regression model which provided significant discriminant power. Additionally, the configuration of intermolecular relationships was distinct between the two donor groups.

The diagnosis of AL amyloidosis requires a tissue biopsy, and diagnostic biomarkers remain an unmet need. Additionally, peripheral blood monoclonal testing is not sensitive enough to detect small levels of amyloidogenic light chains which would continue to cause organ damage. A blood‐based assay has the potential to provide both a readily accessible, objective diagnostic test as well as cellular biomarkers to monitor disease progression.

Our initial findings suggest that a blood‐based diagnostic assay for AL amyloidosis is possible, but more data are required. The most important limitation in our study was the modest sample size with significant differences in gender and race compositions between patients with myeloma and AL amyloidosis. Additionally, the patients were being treated with various agents including daratumumab. Although the study was not powered to consider subgroups, we assessed 17 patients on daratumumab versus 47 patients not treated with daratumumab and found no differences in analyte expression in either cell type with *p* values less than 0.05. Additional studies with a large enough sample size to account for subgroups are warranted. At the same time, the distinction between patients with AL amyloidosis and MGUS and between patients with AL amyloidosis and smouldering myeloma will need to be assessed.

Our efforts have been focused on cell‐type specific molecular expression instead of soluble expression in serum or plasma. Previously, molecular expression levels in the fluid phase have not led to the development of a diagnostic test or biomarkers for patients with AL amyloidosis [28, 29, 30]. Consequently, we thought to access the expression of cell‐associated molecules.

We hypothesised that circulating mononuclear cells have receptors for amyloid deposits and that signalling through those receptors would result in differential expression of signalling pathway molecules. We chose twenty analytes to assess based on that possibility. Although we found both expression level and correlational differences consistent with our hypothesis, these findings need to be validated. Further study will need to show that amyloid deposits initiate signalling in mononuclear cells which results in resolvable expression level and correlational distinctions. Assessing more analytes will provide a more complete picture of the distinct cell‐associated molecular expression associated with AL amyloidosis. Follow‐on studies will be influenced by the identity of the molecules that gave us a signal in our initial study.

BDNF, calmodulin, and phospho‐TBK1 levels were decreased in CD4^+^ T cells from AL amyloidosis patients compared to myeloma patients. Additionally, BDNF and phospho‐TBK1 were tightly correlated in the CD4^+^ T cells from the AL amyloidosis patients but not in the cells from patients with myeloma. BDNF has not been extensively evaluated in lymphocytes, but in cells of the central nervous system in rats, the neurotrophin has been shown to activate calcium/calmodulin‐dependent protein kinase 2 [31, 32]. An inhibitor to Ca^+2^/calmodulin dependent protein kinase kinase β inhibits TBK1 phosphorylation in a murine motor neuron‐like cell line [33], and BDNF and phospho‐TBK1 expression levels in murine brain slices are similarly regulated by exposure to 1‐methyl‐4‐phenyl‐1,2,3,6‐tetrahydropyridine [34]. These findings suggest the possibility of a pathway in human CD4^+^ T cells although the outlines of this pathway are not clearly defined at this point. Our findings suggest that the AL amyloidosis disease process may affect expression levels of these three analytes.

Also, multiple bivariate correlations involving Vav and phospho‐cJun with various phosphoantigens were missing in the lymphocytes from patients with amyloid compared to patients with myeloma. These analytes are known to be essential for the activation of T cells. Vav is known to serve a guanine nucleotide exchange function, which activates Rho family GTPases like Rac1 [35]. Activation of Vav induces cJun phosphorylation through the phosphorylation of JNK (SAPK) by TBK1 [36, 37], which, in turn, phosphorylates cJun.

Although the precise effect of AL amyloid on CD4^+^ T cell physiology cannot be clearly defined from our study, an alteration of the activation pathways in these cells is clear. Because our analysis was not clonally restricted, this effect is not likely dependent on clonal mechanisms but instead involves class‐distributed receptors.

HO1 is an inducible enzyme that acts as an antioxidant and possesses significant anti‐inflammatory activity [38]. In our study, HO1 expression was increased in monocytes from AL amyloidosis patients compared to myeloma patients. The increase did not reach the predetermined threshold (*p* < 0.05), but it was close. Consequently, we included it in logistic regression models and discovered its inclusion markedly improved the predictive power of BDNF expression in CD4^+^ T cells to distinguish between AL amyloidosis patients and myeloma patients without amyloid deposits.

The elevation of HO1 in monocytes associated with the presence of AL amyloid deposits suggests that AL amyloid induces oxidative stress in the monocytes. This interpretation is consonant with the previous finding that amyloid light chains increase oxidative stress in cardiomyocytes [39]. In this previous study, the change in the cellular redox status was indicated by an upregulation of reactive oxygen species as well as an increase in HO1 expression.

Regardless of the mechanism, our findings support the possibility that cell‐associated molecular expression levels can be useful to diagnose disease. Additionally, they may provide prognostic information about disease course and/or response to therapy as well as biomarkers that enhance our understanding of pathogenesis and pathophysiology. This initial analysis sheds light on the potential of cell‐associated molecular expression in patients with AL amyloidosis.

## Author Contributions

**David Kaplan:** conceptualization, methodology, formal analysis, investigation, resources, data curation, writing – original draft, writing – review and editing, funding acquisition. **Eric Christian:** methodology, formal analysis, investigation, data curation, writing – review and editing. **Jason Valent:** resources, writing – review and editing. **Faiz Anwer:** resources, writing – review and editing. **Sandra Mazzoni:** resources, writing – review and editing. **Christy Samaras:** resources, writing – review and editing. **Louis Williams:** resources, writing – review and editing. **Megan Nakashima:** resources, writing – review and editing. **Shahzad Raza:** resources, writing – review and editing. **Mazen Hanna:** resources, writing – review and editing. **Hillard M. Lazarus:** writing – review and editing, funding acquisition. **Jack Khouri:** writing – review and editing, funding acquisition, resources.

## Ethics Statement

All blood donations from patients were conducted according to the guidelines of the Declaration of Helsinki and approved by the Institutional Review Board of the Cleveland Clinic Foundation. Informed consent was obtained from all patients prior to participation.

## Conflicts of Interest

H.M.L. is a paid consultant to Partner Therapeutics, Actinium Pharmaceuticals, CSL Behring, GlycoMimetics Inc., Jazz Pharmaceuticals, Pluristem Therapeutics Inc., and Seattle Genetics. H.M.L. also has an equity position in Partner Therapeutics, is on the speakers' bureau for Jazz Pharmaceuticals and Seattle Genetics and is a member of the Data Safety Monitoring Board for BioSight and for Bristol‐Myers Squibb. The authors have declared no other conflicts of interest exist.

## Supporting information


**Data S1:** jcmm70850‐sup‐0001‐Supinfo.docx.

## Data Availability

The data that support the findings of this study are available from the corresponding author upon reasonable request.
