# Supplementary material for: Molecular Expression Differences in Specific Blood Mononuclear Cell‐Types Identify Patients With AL Amyloidosis
Source: J Cell Mol Med. 2025 Sep 22;29(18):e70850. doi: 10.1111/jcmm.70850 (PMC12451396; doi:10.1111/jcmm.70850)
Supplement: Supplementary file 1 — Data S1: jcmm70850‐sup‐0001‐Supinfo.docx. [file JCMM-29-e70850-s001.docx]

Supplemental Table 1 Description of patients in the study

|  | **Myeloma** | **AL amyloidosis** | **p value** |
| --- | --- | --- | --- |
| **gender** | **male** 19  **female** 21 | **male** 21  **female**  6 | 0.007 |
| **age** | 64.6 (range: 48-81) | 67.7 (range: 51-82) | 0.20 |
| **race** | **African American** 11  **Caucasian** 28  **Asian** 1 | **African American** 2  **Caucasian** 25  **Asian** 0 | 0.02 |
| **treatment** | **treatment** 34  **no treatment** 3  **not specified** 3 | **treatment** 18  **no treatment** 8  **not specified** 1 | 0.99 |

Supplemental Figure 1. Representative histograms for CD4^+^ T cells. Peripheral blood mononuclear cells were stained for CD4 expression, and the signals for the analytes indicated were amplified.

Supplemental Figure 2. Expression level differences in CD4^+^ T cells from patients with myeloma and patients with AL amyloidosis. The p values for the t tests are shown at the bottom of each panel.

Supplemental Figure 3. Expression level differences in monocytes from patients with myeloma and patients with AL amyloidosis. The p values for the t tests are shown at the bottom of each panel.

Supplemental Figure 4. Representative bivariate plots from CD4^+^ T lymphocytes. Correlation coefficients are shown in the lower right corner of each panel. Myeloma patients n = 41. AL amyloidosis patients n = 27.

Supplemental Table 2. Bivariate relationships in CD4^+^ T Lymphocytes

| **keystone molecule** | **molecular partners** | **myeloma** | | **AL amyloidosis** | |
| --- | --- | --- | --- | --- | --- |
|  |  | **r** | **p** | **r** | **p** |
| phospho-p38 MAPK | phospho-RIP | 0.84 | 1x10^-11^ | 0.71 | 3x10^-5^ |
|  | phospho-Shp2 | 0.79 | 1x10^-9^ | 0.77 | 3x10^-6^ |
|  | phospho-SQST | 0.83 | 3x10^-11^ | 0.73 | 2x10^-5^ |
|  | phospho-STING | 0.79 | 1x10^-9^ | 0.90 | 2x10^-10^ |
|  | phospho-ULK1 | 0.76 | 1x10^-8^ | 0.62 | 6x10^-4^ |
| phospho-RIP | phospho-Shp2 | 0.81 | 2x10^-10^ | 0.72 | 2x10^-5^ |
|  | phospho-SQST | 0.86 | 1x10^-12^ | 0.69 | 7x10^-7^ |
|  | phospho-STING | 0.85 | 4x10^-12^ | 0.70 | 5x10^-5^ |
|  | phospho-ULK1 | 0.86 | 1x10^-12^ | 0.70 | 5x10^-5^ |
| phospho-Shp2 | phospho-SQST | 0.90 | 3x10^-15^ | 0.92 | 1x10^-11^ |
|  | phospho-STING | 0.79 | 1x10^-9^ | 0.70 | 5x10^-5^ |
|  | phospho-ULK1 | 0.79 | 1x10^-9^ | 0.60 | 9x10^-4^ |
| phospho-SQST | phospho-STING | 0.80 | 6x10^-10^ | 0.63 | 4x10^-4^ |
|  | phospho-ULK1 | 0.81 | 2x10^-10^ | 0.67 | 1x10^-4^ |
| phospho-STING | phospho-ULK1 | 0.82 | 1x10^-10^ | 0.61 | 8x10^-4^ |

Supplemental figure 5. Schematic depiction of intercorrelating molecules in CD4+ T cells from patients with myeloma and AL amyloidosis.

| **cell-type** | **keystone molecule** | **molecular**  **partners** | **myeloma** | | **AL amyloidosis** | | **r to z (p values)** |
| --- | --- | --- | --- | --- | --- | --- | --- |
|  |  |  | **r** | **p** | **r** | **p** |  |
| CD4^+^ T cells | BDNF | phospho-TBK1 | 0.16 | 0.32 | 0.75 | 7x10^-6^ | 0.0019 |

Supplemental Table 3. Bivariate correlations in AL amyloidosis patients but not in myeloma patients

Correlation coefficients (r) with their associated p values are shown. Values of r were compared between samples from patients with myeloma and AL amyloidosis after conversion to z.

Supplemental Figure 6. Bivariate plots of BDNF and phospho-TBK1 in CD4^+^ T lymphocytes from myeloma and AL amyloidosis patients.

Supplemental Table 4. Bivariate correlations centered on BDNF expression in monocytes

| **keystone molecule** | **molecular**  **partners** | **myeloma** | | **AL amyloidosis** | | **r to z (p values)** |
| --- | --- | --- | --- | --- | --- | --- |
|  |  | **r** | **p** | **r** | **p** |  |
| BDNF | calmodulin | 0.83 | 4x10^-11^ | 0.55 | 3x10^-3^ | 0.030 |
|  | Vav | 0.68 | 1x10^-6^ | 0.32 | 0.10 | 0.0574 |
|  | phospho-ULK1 | 0.73 | 9x10^-8^ | 0.28 | 0.16 | 0.0143 |
|  | phospho-RIP | 0.67 | 2x10^-6^ | 0.30 | 0.10 | 0.0561 |
|  | phospho-SQST | 0.64 | 9x10^-6^ | 0.09 | 0.66 | 0.0108 |

Correlation coefficients (r) with their associated p values are shown. Values of r were compared between samples from patients with myeloma and AL amyloidosis after conversion to z.

Supplemental Figure 7. Examples of differences in bivariate correlation in monocytes between samples from myeloma patients and patients with AL amyloidosis. Correlation coefficients are shown in the lower right corner of each panel.
